# VA's EHR transition and health professions trainee programs: Findings and impacts of a multistakeholder learning community

**DOI:** 10.1002/lrh2.10460

**Published:** 2024-10-23

**Authors:** Julian Brunner, Ellen A. Ahlness, Ekaterina Anderson, Brianne K. Molloy‐Paolillo, Alexandre Braga, Sarah L. Cutrona, Christian D. Helfrich, Deborah Levy, Erin Matteau, Edward Walton, George Sayre, Seppo T. Rinne

**Affiliations:** ^1^ Center for the Study of Healthcare Innovation Implementation & Policy, VA Greater Los Angeles Health Care Los Angeles California USA; ^2^ Seattle‐Denver Center of Innovation for Veteran‐Centered and Value‐Driven Care VA Medical Center Seattle Washington USA; ^3^ Center for Healthcare Organization and Implementation Research VA Bedford Healthcare System Bedford Massachusetts USA; ^4^ Division of Health Informatics & Implementation Science, Department of Population and Quantitative Health Sciences University of Massachusetts Chan Medical School Worcester Massachusetts USA; ^5^ Seattle‐Denver Center of Innovation VA Puget Sound Health Care System Seattle Washington USA; ^6^ Health Systems and Population Health School of Public Health, University of Washington Seattle WA USA; ^7^ Center of Innovation for Pain Research, Informatics, Multimorbidities, and Education (PRIME) VA Connecticut Health Care West Haven Connecticut USA; ^8^ Yale University School of Medicine New Haven Connecticut USA; ^9^ VA Office of Academic Affiliations Washington DC USA; ^10^ University of Washington School of Public Health Seattle Washington USA; ^11^ Division of Pulmonary and Critical Care Medicine Chobanian and Avedisian School of Medicine, Boston University Boston Massachusetts USA

**Keywords:** electronic health record implementation, learning health system, medical education, partnered evaluation, qualitative methods, United States Department of Veterans Affairs

## Abstract

**Introduction:**

The Department of Veterans Affairs (VA) is undergoing an unprecedented electronic health record (EHR) transition, switching from its homegrown EHR to a commercial system. The transition affects nearly every clinical employee but is particularly disruptive to health professions trainees (HPTs)—an often‐overlooked population in EHR transitions. To better understand and address trainee challenges with the EHR transition, we formed a multistakeholder learning community. In this study, we describe the findings of this learning community and the practices and policies developed in response.

**Methods:**

In the qualitative study designed and executed by our learning community, we conducted 51 interviews with HPTs, program leaders, and preceptors before and multiple times after an EHR transition site's go‐live (February 16, 2022 to April 7, 2023). We merged interview transcripts with 125 survey free‐text responses from a survey conducted with preceptors 2 months post‐go‐live and conducted thematic analysis to identify key themes. To complement qualitative findings, we also include a quantitative survey finding, and, where applicable, we note policy and practice responses spurred by our learning community.

**Results:**

Interviews yielded six key themes: (1) High satisfaction with HPT programs, despite negative impacts of the EHR transition; (2) early delays, then substantial improvements, in HPTs' EHR access; (3) persistent challenges with HPTs' EHR training and support, mitigated by local and national efforts; (4) the challenge of learning to use a rapidly evolving EHR during clinical training; (5) reduced visit volume as a continuing barrier to education; and (6) an impression that HPTs' relative lack of exposure to the prior EHR facilitated their proficiency with the new EHR.

**Conclusions:**

Findings highlighted challenges for HPT programs related to the EHR transition, which spurred important changes including the creation of a national VA council to represent the needs of HPTs in the EHR transition, and improvements to HPTs' EHR training and access.

## 1 INTRODUCTION

The Veterans Administration (VA) is in the midst of an unprecedented, enterprise‐wide electronic health record (EHR) transition, switching each of its facilities, one by one, from its homegrown EHR to a commercial system (Cerner/Oracle Health). The transition is expected to occur over the course of 10+ years, at an estimated cost over $50 billion.^1^ This pivotal moment offers a unique opportunity to examine the experience of health professions trainees (HPTs) at sequential stages of the EHR transition.

HPTs (including students, interns, and residents across health professions) are an often‐overlooked population in EHR transitions despite their important role in care delivery.^2^ VA operates the largest education and training platform for health professionals in the nation,^3^ and many VA medical centers rely on HPTs to care for their Veterans. Each year, more than 120 000 HPTs participate in over 7700 training programs offered through partnerships between 150 VA medical facilities and over 1400 academic institutions.^4^, ^5^

In a formative evaluation of frontline staff experiences with VA's first EHR transition site in Spokane, WA, early signals about the EHR transition's unique impact on HPTs became evident, including problems with EHR training, role assignment, and onboarding of HPTs.^6^, ^7^, ^8^, ^9^ These barriers resulted in many HPTs lacking EHR access during the early EHR transition, and academic programs suspending educational relationships with VA facilities. These findings are consistent with the limited prior literature that identified EHR transitions' adverse impacts on HPTs' learning and supervision.^10^, ^11^

To better understand and address HPT program challenges with the EHR transition, we developed a multistakeholder learning community^12^—a critical component of a learning health system—tasked with collaboratively designing an evaluation focused on HPT programs in Columbus, OH (another early EHR transition site), and formulating policy and practice responses. This learning community included the VA's Office of Academic Affiliations (OAA) and facility leaders who each helped shape the evaluation to meet their informational needs and enacted changes based on study findings, paired with a multidisciplinary team of investigators who determined the ultimate design of the evaluation and conducted the data collection and analysis. The learning community was initiated by a Memorandum of Understanding between investigators and OAA, and later recruited participation from facility leaders involved in the EHR transition. Initial findings from this learning community's evaluation in Columbus highlighted some of the challenges encountered in the first 2 months after the site went live with the new EHR, including delays in HPTs' access to the EHR and other impediments to their optimal involvement in care.^13^ However, these initial findings from 2 months after go‐live were not able to capture the longer‐term equilibrium of the site after its early post‐go‐live phase, nor could they convey the policy and practice changes made in response to initial findings.

### 1.1 Questions of interest

The objectives of this article are to characterize the challenges faced by HPT programs during an EHR transition, to track how these challenges evolved over time, and to describe the policy and practice responses developed by our learning community to address these challenges. In doing so, we illustrate a “learning cycle”^12^ within a learning health system.

## 2 METHODS

The evaluation's data collection encompassed interviews and surveys at the VA in Columbus, OH before, during, and after go‐live (February 16, 2022 to April 7, 2023) (Table 1).

**TABLE 1:** Overview of data included in this study.

Data type	Timing relative to go‐live	Participant roles	Number of observations
Qualitative interviews^a^	2 mo pre, 1 mo post, 2 mo post, and 10 month post	HPTs (*n* = 9), HPT supervisors/preceptors (*n* = 8), hospital/university leadership (*n* = 5)	2 mo pre‐go‐live: 16 1 mo post‐go‐live: 15 2 mo post‐go‐live: 9 10 mo post‐go‐live: 11
Qualitative survey responses^b^	2 mo post	HPT supervisors/preceptors	2 mo post‐go‐live: 125
Survey rating scale	2 mo post and 10 mo post	HPT supervisors/preceptors	2 mo post‐go‐live: 111 10 mo post‐go‐live: 135

^a^ Indicated by quotes starting with “I___.”

^b^ Indicated by quotes starting with “S___.”

Abbreviations: HPT, Health Professions Trainee; mo, month.

### 2.1 Sampling and recruitment

After securing support from site leadership, we contacted service leads to identify frontline clinicians, staff, and HPTs for interview recruitment. Snowball sampling identified additional participants across a range of service areas, with an emphasis on two clinical areas with large HPT programs: eye care and mental health. We conducted 51 interviews with 16 HPTs, program leaders, and preceptors at the Columbus VA in multiple waves: 2 months pre‐go‐live (16), 1 month post‐go‐live (15), 2 months post‐go‐live (9), and 10 months post‐go‐live (11). Interviews were ~60 min in duration, with the exception of 10–15 min “check‐ins” conducted 1 month post‐go‐live, when participants' competing demands were especially intense.

### 2.2 Interviews and survey data collection

Interviews were conducted using semi‐structured interview guides. Preceptor interviews covered several aspects of the EHR transition (Appendix S1) while HPT and program lead interviews were explicitly focused on issues of relevance to trainees (Appendix S2).

Both sets of interview guides incorporated questions reflecting a priori and emergent domains of interest, which were pilot‐tested at a prior study site. All interviews were conducted remotely via MS Teams™ by trained qualitative methodologists. During interviews, interviewers used grounded probes (Appendices S1 and S2) to elicit additional information and clarification.^14^ Interviewers completed post‐interview debrief notes summarizing content and emerging reflections, which were discussed among the full evaluation team. Interviews were professionally transcribed, with identifying information removed.

Survey data were collected as part of a survey on the EHR transition experience (Appendix S3) that included two HPT‐relevant items. The first, a qualitative (free‐text) item, asked respondents at 2‐months post‐go‐live who self‐identified as HPT supervisors, “How has the Cerner EHR implementation affected the VA's training mission at your facility?” The second, a quantitative item, asked these same respondents to rate, from “very negatively” to “very positively,” the impact of the EHR implementation on HPTs educational experience at VA (5‐point Likert scale). This quantitative item was repeated at 10 months post‐go live.

### 2.3 Analysis

We conducted thematic analysis to identify themes relevant to the study's questions of interest.^15^ Interview transcripts and survey free‐text responses were coded in ATLAS.ti 23 using a combination of a priori code categories (based on study aims and literature) and emergent codes (with open coding to capture data outside a priori categories). The coding team met weekly to discuss emerging analytical insights. Building upon these insights, the first author reviewed data relevant to the aims of this analysis (all free‐text responses, all quotations from HPTs, and all other quotations coded as relevant to HPTs) to generate themes, which were iteratively reviewed and refined with co‐authors.

## 3 RESULTS

Interviews were consistent with survey responses in conveying an overall negative initial impression of the impact of the transition on HPT programs, which was slightly but incompletely improved by 10 months after go‐live.

Analyses revealed six key themes: (1) High satisfaction with HPT programs, despite negative impacts of the EHR transition; (2) early delays, then substantial improvements, in HPTs' EHR access; (3) persistent challenges with HPTs' EHR training and support, mitigated by local and national efforts; (4) the challenge of learning to use a rapidly evolving EHR during clinical training; (5) reduced visit volume as a continuing barrier to education; and (6) an impression that HPTs' relative lack of exposure to the prior EHR facilitated their proficiency with the new EHR. Each of these themes is explored below using verbatim quotes to illustrate participant perspectives. Quotes are labeled with their wave of data collection (e.g., 2 months before go‐live), and unique participant ID.

### 3.1 High satisfaction with HPT programs, despite negative impacts of the EHR transition

Perspectives about HPT programs at VA were favorable overall, despite challenges related to the EHR transition. Some HPTs indicated that the VA's patient population was a meaningful driver of their satisfaction with training, along with the quality of education and support from supervisors. This helped explain their general satisfaction with their experience despite negative impacts of the EHR transition perceived by HPTs' supervisors. In our survey of self‐identified HPT supervisors at 2 months after go‐live, 82% reported the EHR transition had negatively impacted the VA's training mission, with 70% reporting the same at 10 months.

HPTs who were dissatisfied with the EHR transition overwhelmingly communicated that their experience with the EHR was a relatively minor component of their overall training experience.

“While there are some minor issues with [the EHR transition], it doesn't take away from my job. …**the whole idea is it's taking care of Vets and I'll do that with or without Cerner**” (I213_10mo_post).

A small minority of participants suggested that the new EHR made them somewhat less interested in working at VA, with one HPT attributing this perspective not to the transition process, but to the design of the new system:

“Other EMRs … just capture the nuances of our [ophthalmology] exam a little better, so **I would probably shy away from a place with Cerner**” (I204_2mo_post).

Conversely, another framed the EHR transition as giving HPTs at early transition sites a competitive advantage:

“My hope is to continue to work with the VA. However, the other VAs around the country don't have Cerner, so [my experience with Cerner at VA] would kind of **give me an edge**” (I214_10mo_post).

### 3.2 Early delays, then substantial improvements, in HPTs' EHR access

The most prominent initial obstacle that HPT programs encountered was that, in the early months after go‐live, HPTs were frequently unable to access the new EHR for weeks after the start of their programs. Particularly for shorter programs, this severely limited HPT involvement in care, as noted by some supervisors:

“The **effectiveness of rotations depend on access to the EMR**, and [lags in access]… means a trainee could not have access for the majority of their rotation” (S220_2mo_post).

“[Some] residents **don't get access during their rotation** at all so end up shadowing only” (S137_2mo_post).

This phenomenon had emerged at a prior EHR transition site and was a significant motivation for establishing the learning community described in this study. Barriers to timely access included onerous training requirements, challenges scheduling EHR training, numerous preconditions before the vendor would begin granting access, and a lag in access even after preconditions were met.

In response to these early challenges, the VA's Office of Academic Affiliations—a central member of our learning community—helped institute policies that reduced in‐person training requirements for HPTs (as described in the next theme). It also helped develop training about HPT “provisioning” (EHR role assignment) for the technical staff in charge of coordinating EHR roles for all facility employees, and advised each site to identify a specific coordinator for HPTs' EHR roles. More broadly, the office established a “community of practice” group to engage monthly with HPT stakeholders from sites implementing the new EHR. After these changes, interviews 10 months after go‐live revealed substantial improvements in HPT access:

“**The first set of students that we had after [the EHR] transition, getting them their access was like pulling teeth**. And they had no Cerner access for, like, weeks after they started. [But] the ones that just started in January, they had Cerner access … even before they came … so **it went much better the second time around**” (I212_10mo_post).

One supervisor even suggested that access to the EHR was eventually more timely for HPTs than for permanent employees:

“As soon as [HPTs] get their [identification badge], **within a day or two they will have access to the system** …. Unfortunately, it doesn't happen [as quickly] for our regular staff” (I202_10mo_post).

### 3.3 Persistent challenges with HPTs' EHR training and support, mitigated by local and national efforts

Participants also reported profound initial challenges with training on the new EHR throughout the transition. HPTs and supervisors alike noted that the sheer amount of content covered in training was unduly high for trainees. Many suggested that the training was poorly targeted to the actual responsibilities of each HPT, and “not at all specific to their role in our clinic” (S152_2mo_post).

As with EHR access problems, many of the issues with EHR training were evident at a prior EHR transition site, and many were experienced by HPTs and long‐term employees alike. Informed by data from early rounds of our evaluation, local and national leaders of HPT programs took steps to: (a) reduce vendor‐imposed pre‐rotation training requirements that were found to be of low relevance, (b) move some instructor‐led training to asynchronous training not reliant on instructor availability, (c) supplement vendor‐led training with locally‐developed, VA‐specific reference materials and HPT‐relevant at‐the‐elbow training and support, focusing on specialty‐ and clinic‐specific contexts, and (d) provide administrative support to help HPTs identify and complete required training in advance of their rotations. Institutional support for many of these changes was facilitated by the introduction of a new group (a “National EHR Modernization HPT Council”) within the VA's governing structure for the EHR transition.

“We've **gotten some waivers** for some [training requirements] and they've made some things electronic so that [HPTs] can do [EHR training] prior to their first day” (I202_10mo_post).

“A lot of our departments have **made little cheat walkthroughs**, like how to do this note or how to do that note. So we've been trying to collect those and … get those to the students” (I212_10mo_post).

These efforts were noted and appreciated by participants. However, they did not reverse participant perceptions about training entirely. At 10 months post‐go‐live, some of the challenges observed early on appeared to persist, as described by an HPT:

“The training is directed towards some type of general provider who is perhaps working at the VA full time and in multiple settings … **we had to go through the modules** [such as] how to transition a patient from the ICU to a floor, or how to admit somebody from the emergency department, **which were not things that I ever did or, honestly, will do**” (I209_10mo_post).

### 3.4 The challenge of learning to use a rapidly evolving EHR during clinical training

HPTs' learning was sometimes hampered by the rapidly changing nature of desired EHR procedures and of the EHR itself. Shortly after go‐live, one participant noted that supervisors were often unable to advise HPTs on the appropriate way to use the EHR local and national policies continued to change:

“Trainees are … trying to ask questions that sometimes [we] supervisors don't know the answers to … **We can't say ‘well, [for] this procedure, this is the form that you need to use’ or ‘this is how we want your notes to look’ because no one really knows that yet** … We've all been doing whatever we're doing and then, you know, six months from now, a year from now, they're going to say ‘don't do it that way [anymore]’” (I205_1mo_post).

At 2 months after go‐live, participants described persistent, and in some cases, elevated flux in local policies for using the EHR. One supervisor explained:

“I sent out another email updating ‘this is the actual process’ and then, like the next week, someone throws [in] something else and says ‘no, you got to do it this way,’ … some of the primary care people are like ‘**I don't even read the emails anymore because they change their minds about stuff every day**’” (I201_2mo_post).

The same participant offered an example of the kind of local instruction often necessary to complete basic tasks in the EHR:

“For us to order diabetic socks we have to … choose ‘PSAS, general medical device supply’ … and then just put in the instructions that we want diabetic socks. So instead of just making it easy where you can search ‘sock,’ we have to remember to go under general medical device supply, then type in ‘socks’” (I201_2mo_post).

Another supervisor noted that in addition to quickly evolving local policies and procedures, the EHR itself was changing, describing “extreme changes in [EHR system] function, relying on users being able to adjust on the fly to sporadic updates” (S130_2mo_post).

Although these rapid changes were less‐frequently noted by 10 months after go‐live, they were still reported to occur. Often, the content of the changes was benign, but inadequate preparation for the change was disorienting. One supervisor described sudden changes in workflows for the mental health providers embedded in primary care (i.e., Primary Care Mental Health Integration, or PCMHI):

“PCMHI's workflow changed, and they were not aware of it, so **they came in and logged in one day and it was suddenly different**. … they had to step back a little bit to be able to learn that new workflow” (I205_10mo_post).

### 3.5 Reduced visit volume as a continuing barrier to education

Once HPTs had completed EHR training and obtained access to the new EHR, they sometimes faced reduced patient volumes in their programs as a result of the transition, particularly in the first few months after go‐live. By 10 months, some improvements were noted, but overall reduced efficiency and lower patient volumes were still described. Inefficiency was attributed to features of the new EHR—both its design and its technical characteristics. One supervisor explained:

“[Even after] you're used to using the software, **you hit this limit of how quickly you can navigate through the system** just because the buttons are not as responsive as a locally run application, like [the former EHR] was … so I think there's delays there that will never get better with the current system unfortunately” (I201_10mo_post).

Reduced patient volume meant that HPTs participated in fewer patient encounters and procedures, reducing overall educational opportunities, as noted by a site leader:

“**The clinics aren't back to the volume** that we were seeing [before], so that impacts us as a training program [and] the trainees directly cause they're not seeing as many patients, they're not seeing a variety of pathology. They're not generating as many surgeries and procedures” (I205_10mo_post).

In some cases, reduced encounter and procedure volume put training programs in jeopardy, although participants acknowledged that factors other than the EHR transition were at play:

“From an accreditation standpoint … **the residents have to have a certain number of clinical encounters** … [We] received a citation from the ACGME … related to procedural volume. And that's not solely Cerner's fault obviously. There's a lot at play there including pandemics and whatnot. But I can physically see the decreased volume in procedures” (I205_10mo_post).

### 3.6 HPTs' relative lack of exposure to the prior EHR facilitated their proficiency with the new EHR

Relative to long‐term employees, HPTs' expectations about the new EHR were less influenced by the prior EHR's design and functionality. This was seen as benefiting HPTs, and facilitating their proficiency with the new system.

“A trainee that comes into the facility and they don't have any historical experience using [the former EHR], … **they don't really have this preconceived notion of how things should work, and I think that's probably making things a little bit easier on incoming staff and trainees**” (I201_10mo_post).

“I think it's been helpful that most [trainees]… this is the first medical record a lot of them are seeing…so they are kind of **looking at this all with fresh eyes**…they're saying it's making a little more sense to them” (I212_2mo_post).

In fact, some observed that by 10 months after go‐live, HPTs' proficiency with the new EHR had surpassed their attendings'—not only because of their “young and tech savvy” (I205_10mo_post) status, but also because in some clinics, HPTs interact with the EHR more frequently than their attendings:

“The residents are way more comfortable with the EHR at this point. They have used it more than all of us … **The trainees are really more the fount of knowledge than the faculty are at this point**” (I205_10mo_post).

## 4 DISCUSSION

Our learning community identified key themes related to the impact of an EHR transition on HPT programs. Promising findings included positive educational experiences despite EHR challenges, improvements in the timeliness of EHR access for HPTs, and successful local and national efforts to improve HPTs' EHR training through locally created content and peer support. Findings that warrant continued attention during future EHR transition efforts include persistent challenges with training, as well as the difficulty of adapting to a constantly evolving EHR system. Reduced visit volume even at 10 months post‐go‐live was also an important consideration for HPTs who must achieve required competencies that can be influenced by patient volume.

### 4.1 Implications for EHR transitions

In a review of methodologies for evaluating health IT, Kaplan^16^ proposed that “fit”—for example, between an EHR and the organization it will be used in—is a key factor in successful implementation. Aarts et al.^17^ built upon this proposition and added that “fit” is *not* a static characteristic. Rather, they insist, “fit has to be actively produced: the technology and the practice have to be *made* to fit.”

By actively eliciting ways in which current practice and the new technology were misaligned, and catalyzing policy and technology changes in response, this study serves as a model for the process of “actively producing” fit. It involves close attention to the ways in which needs of HPTs both mirror and diverge from those of other EHR users and others affected by the EHR transition. Just as implementers of medical interventions must take care to consider heterogenous responses from varied patient subpopulations, EHR transitions affect subgroups of employees with starkly different needs that must be carefully attended to in the preparation, configuration, and testing of the EHR. The challenges we document in this study, as well as the promising practice and policy responses we fostered, speak to the necessity of developing a detailed understanding of the diverse subgroups of employees, such as HPTs, who will be affected by an EHR transition, and carefully monitoring them for unanticipated needs related to a new EHR.

### 4.2 A learning health system in action

In addition to implications for EHR transitions, our study serves as a demonstration of a learning health system in action, embodying the three learning cycle stages proposed by Friedman et al.: (1) performance to data, (2) data to knowledge, and (3) knowledge to performance.^12^, ^18^, ^19^

#### 4.2.1 Performance to data

The design and scope of the evaluation were determined by a learning community including OAA, facility leaders, and a multidisciplinary team of investigators. In describing what differentiates learning health systems from other improvement efforts, Friedman (2022) explained that “members of the multistakeholder learning community that drives a learning cycle begin their work from a position of uncertainty … [undertaking] a process of exploration, not confirmation.”^12^ We embraced this principle by beginning with local data collection rather than with the implementation of policies whose rationale stems from external evidence alone.

#### 4.2.2 Data to knowledge

Guided by the learning community and the interests and evidence needs that it identified, we conducted both rapid analyses (for iterative updates) and in‐depth analyses, linking these analyses with external evidence about EHR implementation best practices and emerging evidence about the VA's EHR transition generated by other projects.^20^, ^21^, ^22^, ^23^ We also conducted a focus group with OAA and Columbus facility leadership to review and contextualize preliminary findings and jointly develop actionable policy recommendations.

#### 4.2.3 Knowledge to performance

Most importantly, our findings were translated into policy and practice changes at several levels of the organization, including the creation of a national EHR Modernization HPT Council to elevate the voice of HPTs in decisions about the configuration and implementation of the EHR; the release of a national executive decision memorandum reducing redundant EHR training and making training more accessible; and the introduction of HPT‐tailored EHR support resources.

### 4.3 Limitations

Our study was subject to limitations. While we present findings from multiple time points and note changes over time, our sample was not entirely longitudinal. Not all interviewees participated in all four waves of interviews, and survey free‐text responses were only available from a single wave of data collection (2 months after go‐live). Interview and survey data are subject to potential selection bias, and those who chose to participate in surveys or interviews may not be representative of the full population. Also, our limited interview sample, which emphasizes perspectives from the participating facility's largest training programs, may not encompass the diversity of perspectives from HPT programs across clinical areas. We sought to mitigate this limitation by including open‐ended survey responses from a facility‐wide survey of preceptors.

There are also limitations in our ability to directly assess the policy and practice changes described in our study. This study was not a purpose‐built evaluation of policies enacted by our learning community. We describe initial findings, policy responses, and subsequent findings, but the focus of the formative evaluation was on the impact of the EHR transition on HPT programs, not on the policies that emerged from our work. Similarly, the policy responses we describe happened gradually, with the involvement of institutional actors at several levels of the organization. Our learning community cannot claim exclusive responsibility for their development, and changes we observed across waves of data collection may not be attributable to the policy responses we advanced.

## 5 CONCLUSION

In summary, our partnered evaluation highlighted challenges encountered by HPT programs early in the EHR transition process and helped spur and document improvements. By elucidating diverse experiences at multiple points in the transition, this evaluation generated actionable insights to support the next generation of healthcare professionals amidst a disruptive organizational transformation.

## FUNDING INFORMATION

This work was supported by funding from the US Department of Veterans Affairs, Veterans Health Administration, Health Services Research & Development Quality Enhancement Research Initiative (QUERI) (PEC 21‐280; PEC 21‐168) and the Office of Academic Affiliations. DL receives fellowship funding from the Department of Veterans Affairs, Veterans Health Administration, Office of Academic Affiliations, Office of Research and Development, with resources and the use of facilities at the VA Connecticut Healthcare System, West Haven, CT. (CIN‐13‐407).

## CONFLICT OF INTEREST STATEMENT

The authors declare no conflicts of interest.

## Supporting information


**Appendix S1.** Preceptor interview guides.

**Appendix S2.** HPT and site leader interview guides.

**Appendix S3.** Survey methods and quantitative data.
