# Supplementary material for: VA's EHR transition and health professions trainee programs: Findings and impacts of a multistakeholder learning community
Source: Learn Health Syst. 2024 Oct 23;9(2):e10460. doi: 10.1002/lrh2.10460 (PMC12000766; doi:10.1002/lrh2.10460)
Supplement: Supplementary file 1 — Appendix S1. Preceptor interview guides. [file LRH2-9-e10460-s002.docx]

# Appendix 1. Preceptor Interview Guides

Pre-Implementation Interview Guide

| ***Grounded probes/prompts:*** *If responses are limited or require clarification, probes may be used to elicit more detailed responses. Probes should use words or phrases presented by the participant using one of the following formats:*    *What do you mean by ____________?*  *Tell me more about___________.*  *Give me an example of ____________.*  *Tell me about a time when ____________.*  *Who ____________?*  *Where ____________?*  *What, if anything, was helpful about ________________?*  *What, if anything, was not helpful about ________________?*  *What, if anything, made ________________ difficult?*  *What, if anything, made ________________ easier?*  *What was the impact of ____________?*  *Walk me through __________.*  *How _______________?*  *Tell me about the __________ training session you participated in.* |
| --- |

**Role**

- To start, can you tell me about your role at VA?
- Do you supervise trainees as part of your job? [What types?]
- Have you had any role in the EHR transition?

**Attitudes toward Cerner *(baseline attitudes)***

- In your opinion, how is the CERNER implementation going?
- What concerns do you have about transitioning to CERNER?

**Information**

- Tell me about communication and messaging about the transition

*AS NEEDED:*

- …from local leaders [e.g., chief or supervisor]
- …from national VA leadership

**Preparations for the EHR transition**

- Tell me about preparations for the EHR transition at your facility.
- Have there been any staffing changes or resources to help maintain access during the transition?

**EHR training and education**

- Tell me about any training related to the EHR transition at your facility.

**HPTs**

We’re also interested in how the transition is affecting residents and other health professions trainees (HPTs)

- Have you seen any ways that the transition has affected trainees?
  - [If so,] has the trainee experience with the transition affected you in any way?
- [If applicable] Has the transition affected your responsibilities as a preceptor?

**Conclusion**

- Is there anything else you want us to know?

2-months Post-Implementation Interview Guide

| ***Grounded probes/prompts:*** *If responses are limited or require clarification, probes may be used to elicit more detailed responses. Probes should use words or phrases presented by the participant using one of the following formats:*    *What do you mean by ____________?*  *Tell me more about___________.*  *Give me an example of ____________.*  *Tell me about a time when ____________.*  *Who ____________?*  *Where ____________?*  *What, if anything, was helpful about ________________?*  *What, if anything, was not helpful about ________________?*  *What, if anything, made ________________ difficult?*  *What, if anything, made ________________ easier?*  *What was the impact of ____________?*  *Walk me through __________.*  *How _______________?*  *Tell me about the __________ training session you participated in.* |
| --- |

**Overview**

- Tell me about your overall experience transitioning to Cerner.

**Information/Communication**

- Tell me about communication and messaging regarding Cerner before and after go-live.
  - *Probe:* Chief or supervisor; VISN and VAMC-level leadership; VA national leadership; Cerner
- Tell me what it’s been like to work with your coworkers during the transition.

**Training and education**

- Did the training you received prior to go-live prepare you to use Cerner?
- Have you received any additional training since the go-live? Please tell me about it.
  - [If no]: Would you want to receive more training in using Cerner? Why or why not?
  - [If yes]: Describe the training format. What was most helpful? Least helpful?

**HPTs**

- We’re also interested in how the transition is affecting residents and other health professions trainees. Have you seen any ways that the transition has affected trainees?
  - [If yes]: Has the trainee experience with the transition affected you in any way?
- [If applicable]: Has the transition affected your responsibilities as a preceptor?

**Resources and capacity**

- Tell me about clinic capacity and access for Veterans since go-live
  - *Probe*: NESSU (National EHRM Supplemental Staffing Unit); Clinical Resource Hub; Other resources?

**Using Cerner**

- Tell me about using Cerner.
- Which functions/elements of Cerner do you use on an average day?
- How is it to use Cerner for your job?
- Have you made any modifications or changes to Cerner to fit your work needs better?
- Do you document outside of your regular work hours?
- Tell me about communicating with your team and other coworkers within Cerner.
- We’ve heard that Cerner has different functions for different roles. Has that affected you at all?*Probe*: Is there anything you can no longer do because of these role-based changes? Has it impacted how you work with your colleagues?
- Have anyone’s duties or roles changed because of the EHR transition?

**Support**

- When you need help with something in Cerner, who do you ask?

*Probe*: Helpdesk/helpline? Superusers? In-person support from Cerner? Other online support?

- Tell me about requesting changes to Cerner.
- Tell me about anything your team or clinic did as a group to help you use Cerner.

**Veteran Impact**

- Has the Cerner transition impacted Veterans?

*Probe*: Veteran care? Veteran experience? Patient portal? Patient safety?

**Conclusion**

- What else do you want us to know that I have not asked about?

## 10-month Post-Implementation Interview Guide

| ***Grounded probes/prompts:*** *If responses are limited or require clarification, probes may be used to elicit more detailed responses. Probes should use words or phrases presented by the participant using one of the following formats:*    *What do you mean by ____________?*  *Tell me more about___________.*  *Give me an example of ____________.*  *Tell me about a time when ____________.*  *Who ____________?*  *Where ____________?*  *What, if anything, was helpful about ________________?*  *What, if anything, was not helpful about ________________?*  *What, if anything, made ________________ difficult?*  *What, if anything, made ________________ easier?*  *What was the impact of ____________?*  *Walk me through __________.*  *How _______________?*  *Tell me about the __________ training session you participated in.* |
| --- |

**Overview**

- Tell me about your overall experience transitioning to Cerner.
  - *Probe* on key issues raised in prior interviews
  - *Probe* on changes since the last interview

**Information/Communication**

- Tell me about communication and messaging regarding Cerner since we last spoke.
  - *Probe:* Chief or supervisor; VISN and VAMC-level leadership; VA national leadership; Cerner

**Training and education**

1. Since we last spoke, have you found anything that has helped you with Cerner?
2. Have you received any additional training since we last spoke? Please tell me about it.
   - *[If no]: Would you want to receive more training in using Cerner? Why or why not?*

**HPTs**

- We’re also interested in how the transition is affecting residents and other health professions trainees. More recently, have you seen any ways that the transition has affected trainees?
  - [If yes]: Has the trainee experience with the transition affected you in any way?
- [If applicable]: Has the transition affected your responsibilities as a preceptor?

**Resources and capacity**

- Tell me about clinic capacity and access for Veterans since we last spoke
  - *Probe*: NESSU (National EHRM Supplemental Staffing Unit); Clinical Resource Hub; Other resources?

**Using Cerner**

- Tell me about using Cerner now that it’s been in place for a while longer.
- Have you made any modifications or changes to Cerner to fit your work needs better?
- Tell me about communicating with your team and other coworkers within Cerner.
- We’ve heard that Cerner has different functions for different roles. Has that affected you at all?
  - *Probe*: Has that changed in any way since our last interview?
- Have anyone’s duties or roles changed because of the EHR transition?

**Support**

- When you need help with something in Cerner, who do you ask?

*Probe*: Helpdesk/helpline? Superusers? Other online support?

- Tell me about requesting changes to Cerner.
- Tell me about anything your team or clinic did as a group to help you use Cerner.
- Tell me about working with or learning from other sites.

**Veteran Impact**

- Has the Cerner transition impacted Veterans?

**Patient Safety**

- Tell me about any patient safety concerns since we last spoke.

**Conclusion**

- What else do you want us to know that I have not asked about?
- [Time permitting] What advice would you give to any of the sites scheduled to transition next?
