# Supplementary material for: VA's EHR transition and health professions trainee programs: Findings and impacts of a multistakeholder learning community
Source: Learn Health Syst. 2024 Oct 23;9(2):e10460. doi: 10.1002/lrh2.10460 (PMC12000766; doi:10.1002/lrh2.10460)
Supplement: Supplementary file 2 — Appendix S2. HPT and site leader interview guides. [file LRH2-9-e10460-s003.docx]

# Appendix 2. HPT & Site Leader Interview Guides

## Pre-Implementation Interview Guide

| ***Grounded probes/prompts:*** *If responses are limited or require clarification, probes may be used to elicit more detailed responses. Probes should use words or phrases presented by the participant using one of the following formats:*    *What do you mean by ____________?*  *Tell me more about___________.*  *Give me an example of ____________.*  *Tell me about a time when ____________.*  *Who ____________?*  *Where ____________?*  *What, if anything, was helpful about ________________?*  *What, if anything, was not helpful about ________________?*  *What, if anything, made ________________ difficult?*  *What, if anything, made ________________ easier?*  *What was the impact of ____________?*  *Walk me through __________.*  *Tell me about the __________ training session you participated in.* |
| --- |

### Site Leader-specific questions

**HPT’s role, background, trajectory (and a bit about their program)**

- Tell me about the [training program] and your role.

*AS NEEDED:*

- How long is the program and when did it start?
- How many hours do you train at the VA each week?
- What are your duties and responsibilities in the program?
- Tell me about the clinical settings and teams that you work with at VA.
- Tell me how mentorship and supervision works in the program.
- Tell me about how your time at VA fits into your training trajectory

**Communication and training**

- Did you receive any information about VA’s transition to Cerner before starting your VA training?
- Tell me about any training you’ve received for using Cerner at VA.
- How have expectations about the Cerner transition impacted your training experience (if at all)?
- How, if at all, has the transition to Cerner impacted your training experience?
  - *AS NEEDED: probe for examples.*

*AS NEEDED*:

- What did you find most helpful about the training?
- Is there anything you wish you’d learned more about in the training?
- Before starting your VA rotation, how much time did you spend in EHR training?
- Have you had any opportunities to practice with the system on your own?

**Prior EHR experience**

- What EHR experience did you have prior to your VA training?

*AS NEEDED:*

- Do you have experience transitioning to a new EHR?

**Plans**

- Have you considered working for the VA following your training?
- Did your experience with the EHR transition impact your decision to work for VA in the future?

### HPT-specific questions

**HPT’s role, background, trajectory (and a bit about their program)**

- Tell me about the [training program] and your role.

*AS NEEDED:*

- How long is the program and when did it start?
- How many hours do you train at the VA each week?
- What are your duties and responsibilities in the program?
- Tell me about the clinical settings and teams that you work with at VA.
- Tell me how mentorship and supervision works in the program.
- Tell me about how your time at VA fits into your training trajectory

**Communication and training**

- Did you receive any information about VA’s transition to Cerner before starting your VA training?
- Tell me about any training you’ve received for using Cerner at VA.
- How have expectations about the Cerner transition impacted your training experience (if at all)?
- How, if at all, has the transition to Cerner impacted your training experience?
  - *AS NEEDED: probe for examples.*

*AS NEEDED*:

- What did you find most helpful about the training?
- Is there anything you wish you’d learned more about in the training?
- Before starting your VA rotation, how much time did you spend in EHR training?
- Have you had any opportunities to practice with the system on your own?

**Prior EHR experience**

- What EHR experience did you have prior to your VA training?

*AS NEEDED:*

- Do you have experience transitioning to a new EHR?

**Plans**

- Have you considered working for the VA following your training?
- Did your experience with the EHR transition impact your decision to work for VA in the future?

### All participants

**Conclusion**

- Is there anything else you want us to know?
- Is there anyone else you’d recommend we talk to?

## 1-Month Post Interview Guide (Check-ins)

| ***Grounded probes/prompts:*** *If responses are limited or require clarification, probes may be used to elicit more detailed responses. Probes should use words or phrases presented by the participant using one of the following formats:*  *What do you mean by ____________?*  *Tell me more about___________.*  *Give me an example of ____________.*  *Tell me about a time when ____________.*  *Who ____________?*  *Where ____________?*  *What, if anything, was helpful about ________________?*  *What, if anything, was not helpful about ________________?*  *What, if anything, made ________________ difficult?*  *What, if anything, made ________________ easier?*  *What was the impact of ____________?*  *Walk me through __________.*  *How _______________?*  *Tell me about the __________ training session you participated in.* |
| --- |

- Tell me about how things have been since we spoke last.
- Tell me about transitioning to Cerner.
- Has the Cerner transition impacted vets?

*AS NEEDED:*

- What about Veteran care?
- What about Veteran experience?
  - How is the Cerner transition affecting the patient portal?

**Information/Communication**

- Tell me about communication regarding Cerner since go-live.

*AS NEEDED:*

- Tell me about any information you have received [from local leaders, from chief or supervisor, VA leadership, Cerner]

**Conclusion**

- Is there anything else you want us to know?
- Is there anyone else you’d recommend we talk to?

## 2-Month Post Interview Guide

| ***Grounded probes/prompts:*** *If responses are limited or require clarification, probes may be used to elicit more detailed responses. Probes should use words or phrases presented by the participant using one of the following formats:*    *What do you mean by ____________?*  *Tell me more about___________.*  *Give me an example of ____________.*  *Tell me about a time when ____________.*  *Who ____________?*  *Where ____________?*  *What, if anything, was helpful about ________________?*  *What, if anything, was not helpful about ________________?*  *What, if anything, made ________________ difficult?*  *What, if anything, made ________________ easier?*  *What was the impact of ____________?*  *Walk me through __________.*  *How _______________?*  *Tell me about the __________ training session you participated in.* |
| --- |

**Updates in Training Environment**

- Tell me about how things have been since we spoke last. What recent developments have happened?
- Tell me about transitioning to Cerner. How has the EHR transition impacted you in your role?
- How has the Cerner transition affected your team?
- Has the Cerner transition impacted vets?

*AS NEEDED:*

- What about Veteran care?
- What about Veteran experience?
  - How is the Cerner transition affecting the portals you use?

**Function**

- Walk me through getting access to and using Cerner.

*AS NEEDED:*

- Which functions or elements of Cerner do you typically use?
- When you need help with something in Cerner, who do you ask?

**Information/Communication**

- Tell me about communication regarding Cerner since go-live.

*AS NEEDED:*

- Tell me about any information you have received from [local leaders, from chief or supervisor, VA leadership, Cerner]

**Conclusion**

- What, if any, recommendations do you have regarding EHRM for sites that will go through this process in the future?
- Is there anything else you want us to know?

## 10-Month Post Interview Guide

| ***Grounded probes/prompts:*** *If responses are limited or require clarification, probes may be used to elicit more detailed responses. Probes should use words or phrases presented by the participant using one of the following formats:*    *What do you mean by ____________?*  *Tell me more about___________.*  *Give me an example of ____________.*  *Tell me about a time when ____________.*  *Who ____________?*  *Where ____________?*  *What, if anything, was helpful about ________________?*  *What, if anything, was not helpful about ________________?*  *What, if anything, made ________________ difficult?*  *What, if anything, made ________________ easier?*  *What was the impact of ____________?*  *Walk me through __________.*  *How _______________?*  *Tell me about the __________ training session you participated in.* |
| --- |

### Site-Leader specific questions

**EHRM impact on training experience**

- We’re interested in hearing about how the VA’s new EHR is impacting HPTs and training programs. Can you tell me about anything that has changed since our last interview?
- Has the VA transition to Cerner impacted [trainee’s] experience?
- Has the transition affected your work with VA trainees, or affected the program in other ways?

**EHR access and use**

- Can you walk me through the process of [trainees] getting access to and using Cerner?

*AS NEEDED:*

- Tell me about any EHR training that [trainees] receive.
- What, if any, recommendations do you have regarding EHR training for [trainees]?

**Impact on care**

- Has the transition to Cerner impacted the way trainees/residents provide care to Veterans?

**EHR support**

- We’re also interested in how trainees get help with the EHR when they need it. Do you have any recommendations about improving trainees/residents’ experience with EHR support?

**Conclusion**

- What, if anything, has been helpful in the current trainee EHR transition experience?
- What would you change to make this process better?

### HPT-specific questions

**HPT’s role, background, trajectory (and a bit about their program)**

- Tell me about the [training program] and your role at VA.

*AS NEEDED:*

- When did your training program/rotation start?

**Communication and training**

- Did you receive any information about VA’s transition to Cerner before starting your VA training?
- Tell me about any training you’ve received for using Cerner at VA.

*AS NEEDED*:

- What did you find most helpful about the training?
- Is there anything you wish you’d learned more about in the training?
- Before starting your VA rotation, how much time did you spend in EHR training?
- Walk me through getting access to and using Cerner.

*AS NEEDED:*

- How soon after starting your rotation were you able to access Cerner and see patients?
- Which functions or elements of Cerner do you typically use?
- When you need help with something in Cerner, who do you ask?

**Prior EHR experience**

- What EHR experience did you have prior to your VA training?

*AS NEEDED:*

- Do you have experience transitioning to a new EHR?

**Veteran care**

- Has the Cerner transition impacted veterans?

*AS NEEDED:*

- What about Veteran care?
- What about Veteran experience?

**Plans**

- Tell me about how your time at VA fits into your training trajectory.
- Have you considered working for the VA following your training?
- Did your experience with the EHR impact your interest in working for VA in the future?

### All participants

**Conclusion**

- Is there anything else you want us to know?
- Is there anyone else you’d recommend we talk to?
