# Supplementary material for: VA's EHR transition and health professions trainee programs: Findings and impacts of a multistakeholder learning community
Source: Learn Health Syst. 2024 Oct 23;9(2):e10460. doi: 10.1002/lrh2.10460 (PMC12000766; doi:10.1002/lrh2.10460)
Supplement: Supplementary file 3 — Appendix S3. Survey methods and quantitative data. [file LRH2-9-e10460-s001.docx]

## Appendix 3: Survey methods and quantitative data

### Sampling and recruitment

We distributed a survey to ~1,770 Columbus employees. 458 (25.9%) responded to the 2-month post-go-live survey, and 492 (27.7%) responded to the 10-month post-go-live survey. This study presents findings among the subset who self-identified as HPT supervisors (n=111 at 2 months and n=135 at 10 months post-go-live).

### Survey data collection

Columbus employees were emailed survey invitations with survey links and three follow-up reminder emails. The voluntary, anonymous surveys took ~10 minutes to complete. The surveys covered a wide range of transition experiences and included two targeted questions about the EHR transition’s effect on VA’s training mission. In this HPT-training-focused inquiry we focus on responses from self-reported trainee supervisors, including survey questions informed by emergent themes from pilot interviews with VA HPT supervisors and site leaders from the first go-live site. This included the question “How has the Cerner EHR implementation affected health professions trainees’ educational experience at VA?” Responses were measured on a 5-point Likert scale spanning from "very negatively" to "very positively."

### Analysis

We conducted descriptive analysis, using Microsoft excel, of survey data by identifying the frequencies and proportions of each response, and by aggregating the two least favorable responses (“somewhat negatively” or “very negatively”). We chose this approach because our evaluation was primarily concerned with understanding and mitigating negative impacts of the transition. To reduce the chance that observed differences in study responses were largely attributable to changes in participant composition by wave, we examined participant characteristics at each wave.
